# The Effect of Cryoprotectants and Storage Conditions on the Transfection Efficiency, Stability, and Safety of Lipid‐Based Nanoparticles for mRNA and DNA Delivery

**DOI:** 10.1002/adhm.202203022

**Published:** 2023-03-21

**Authors:** Konstantinos N. Kafetzis, Natalia Papalamprou, Elisha McNulty, Kai X. Thong, Yusuke Sato, Aleksandr Mironov, Atul Purohit, Philip J. Welsby, Hideyoshi Harashima, Cynthia Yu‐Wai‐Man, Aristides D. Tagalakis

**Affiliations:** ^1^ Department of Biology Edge Hill University Ormskirk L39 4QP UK; ^2^ Faculty of Life Sciences & Medicine King's College London London SE1 7EH UK; ^3^ Faculty of Pharmaceutical Sciences Hokkaido University Kita‐12, Nishi‐6, Kita‐ku Sapporo 060–0812 Japan; ^4^ Electron Microscopy Core Facility (RRID: SCR_021147) Faculty of Biology Medicine and Health University of Manchester Manchester M13 9PT UK; ^5^ Oncology Drug Discovery & Women's Health Group Department of Metabolism Digestion & Reproduction Imperial College London London W12 0HS UK; ^6^ Medical School Edge Hill University Ormskirk L39 4QP UK

**Keywords:** cryoprotectants, DNA, lipid nanoparticles, mRNA, stability

## Abstract

Lipid‐based nanoparticles have recently shown great promise, establishing themselves as the gold standard in delivering novel RNA therapeutics. However, research on the effects of storage on their efficacy, safety, and stability is still lacking. Herein, the impact of storage temperature on two types of lipid‐based nanocarriers, lipid nanoparticles (LNPs) and receptor‐targeted nanoparticles (RTNs), loaded with either DNA or messenger RNA (mRNA), is explored and the effects of different cryoprotectants on the stability and efficacy of the formulations are investigated. The medium‐term stability of the nanoparticles was evaluated by monitoring their physicochemical characteristics, entrapment and transfection efficiency, every two weeks over one month. It is demonstrated, that the use of cryoprotectants protects nanoparticles against loss of function and degradation in all storage conditions. Moreover, it is shown that the addition of sucrose enables all nanoparticles to remain stable and maintain their efficacy for up to a month when stored at −80 °C, regardless of cargo or type of nanoparticle. DNA‐loaded nanoparticles also remain stable in a wider variety of storage conditions than mRNA‐loaded ones. Importantly, these novel LNPs show increased GFP expression that can signify their future use in gene therapies, beyond the established role of LNPs in RNA therapeutics.

## Graphical abstract

In this study, the impact of storage conditions and the use of cryoprotectants on the medium‐term preservation of lipid‐based nanocarriers is investigated. Nanoparticle stability appears to be directly related to temperature, use of cryoprotectants and type of encapsulated nucleic acid cargo. The effect of storage on the physicochemical characteristics, entrapment efficiency, and transfection efficiency of the nanocomplexes is presented here.

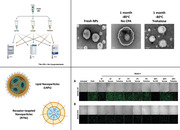

## 1 Introduction

Over the last 20 years, lipid‐based nanoparticles have been increasingly used as the preferred method of delivery, in order to safely and efficiently introduce a variety of novel therapeutics (DNA, mRNA, siRNA, chemical compounds/drugs) in vitro and in vivo, demonstrating very promising results.^[^ ^1^, ^2^, ^3^, ^4^, ^5^, ^6^, ^7^, ^8^, ^9^ ^]^ More recently, lipid nanoparticles (LNPs) were successfully used in both COVID‐19 mRNA vaccines (i.e., Pfizer‐BioNTech^[^ ^10^ ^]^ and Moderna^[^ ^11^ ^]^) that have received Food and Drug Administration (FDA) approval for use even in children under the age of 5,^[^ ^12^ ^]^ demonstrating once again the versatility, safety, and efficacy of this technology.^[^ ^13^ ^]^ However, in the early days of the vaccine roll‐out, the lack of information regarding their stability and the extreme storage conditions required (−80 °C), proved to be the biggest challenge, often causing logistical complications and the waste of precious vaccines, since many healthcare providers decided to be overly cautious.^[^ ^14^ ^]^

Stability is an integral part of the transition of novel therapeutics from bench to clinic, and lipid‐based nanoparticles are no exception. Many studies have concentrated over the years on improving their efficiency and safety profiles,^[^ ^15^, ^16^ ^]^ including improving nucleic acid stability, for example, through mRNA modifications.^[^ ^17^, ^18^, ^19^, ^20^ ^]^ Nevertheless, with many formulations now approaching their final stage before approval,^[^ ^21^ ^]^ the best conditions for nanoparticle (NP) storage, including temperature, physical state, and the use of cryoprotectants, as well as their effect on nanoparticle stability and efficacy, remain poorly understood. Moreover, in addition to aiding the transition from bench to bedside, identifying the optimal storing conditions can benefit everyday researchers by providing convenience, consistency, and reproducibility to their experiments.^[^ ^22^ ^]^

When using the term “nanoparticle stability”, we generally refer to the preservation of the physicochemical characteristics (i.e., size, charge) over a set period of time.^[^ ^23^ ^]^ Nanoparticles self‐assemble due to hydrophobic–hydrophilic, as well as electrostatic, interactions between their different components, and even though this process results in robust, tightly packed formulations, they are inevitably destined to become unstable and break apart over time due to their thermodynamically unfavorable state.^[^ ^22^, ^23^ ^]^ There are two distinct mechanisms involved in NP instability, mechanical stress, which can lead to damage of the liposomal membranes, and chemical transformations, that can potentially influence the in vivo fate of the NPs by affecting their loading and releasing properties.^[^ ^24^, ^25^ ^]^ Some of the most common issues include the hydrolysis or oxidation of phospholipids, the aggregation and fusion of the nanoparticles, as well as the gradual permeabilization of their membranes, which can result in leaky NPs.^[^ ^26^, ^27^, ^28^, ^29^ ^]^

Consequently, in order to extend NP shelf‐life and promote their medium/long‐term stability, some strategies commonly enlisted rely on the use of stabilizing agents, such as polyethylene glycol (PEG)^[^ ^13^ ^]^ (i.e., PEGylation of lipids), cryoprotectants (e.g., sucrose, trehalose, mannitol, etc.),^[^ ^26^, ^30^ ^]^ and the production of dry liposomal products by means of lyophilization (i.e., freeze‐drying).^[^ ^31^, ^32^ ^]^ However, even though lyophilization is a common practice in industry for the large‐scale production of therapeutic products, including NPs, the equipment required is not readily available to most laboratories and researchers, and additionally, the optimization of the process can be very time‐consuming and labor intensive.^[^ ^33^ ^]^

Cryoprotectant agents (CPAs), and specifically sugars, are being widely used in a variety of biological applications, since they are biologically safe and can promote the cryopreservation of tissues, cells, and NPs in sub‐zero conditions over long periods of time.^[^ ^34^ ^]^ Of note, both authorized COVID‐19 mRNA vaccines requiring storage in freezing conditions, include sucrose (10% w/v), a disaccharide and well‐known cryoprotectant, in their list of ingredients.^[^ ^6^, ^13^, ^35^ ^]^ Incidentally, trehalose, another disaccharide and a CPA, is also found in the tissues of tardigrades (water bears), where it supports their survival under extreme temperatures.^[^ ^36^ ^]^

Currently, the means by which CPAs exert their cryoprotective effects are unknown, however, there are a few proposed theories explaining their mechanism of action. In brief, the water replacement theory suggests that sugars bind to the exposed polar head groups of the NP lipids, stabilizing them, whereas the vitrification model hypothesizes that the addition of sugars creates a highly viscous, glass matrix around the NPs, preventing aggregation and formation of ice crystals.^[^ ^22^, ^26^ ^]^ Another theory, the particle isolation hypothesis, argues that the addition of sugars can increase the volume of the unfrozen fraction, keeping individual NPs from interacting.^[^ ^37^ ^]^ Regardless of the mechanism of action, the stabilization effect of CPAs appears to be concentration dependent,^[^ ^22^ ^]^ with concentrations between 10% and 20% w/v generally demonstrating better cryoprotection,^[^ ^22^, ^38^, ^39^, ^40^ ^]^ although long‐term stability has also been achieved with lower concentrations.^[^ ^26^, ^32^, ^37^ ^]^

In the present study, we explore the effect of temperature (i.e., room temperature (RT), 4 °C, −80 °C) and cryoprotectants (i.e., no CPA, 12% w/v sucrose, 12% w/v trehalose) on the stability and efficacy of NPs (Figure S1A, Supporting Information), in order to determine the best storage conditions while maximizing convenience, consistency, and reproducibility. To that end, we synthesized and tested 2 distinct types of lipid‐based nanocarriers; Receptor‐targeted nanocomplexes (RTNs), which comprised of liposome, targeting peptide and nucleic acid, and LNPs utilizing a pH‐sensitive lipid to encapsulate nucleic acids. Both types of NPs have previously demonstrated exceptional transfection efficiencies and low cytotoxicity, by our group and others,^[^ ^41^, ^42^, ^43^, ^44^, ^45^, ^46^, ^47^, ^48^, ^49^, ^50^, ^51^, ^52^, ^53^ ^]^ thus presenting ideal models for our current and future research. Additionally, a peptide targeting the low‐density lipoprotein receptor (LDLR), peptide AT‐19, was used for the formulation of the LNPs, as it has been previously suggested that the addition of targeting peptides can increase the encapsulation efficiency and overall stability of the LNPs.^[^ ^46^ ^]^ Throughout this study, NP stability was evaluated by monitoring their physicochemical characteristics, DNA/mRNA encapsulation, and transfection efficiency, through a variety of methods and assays. Moreover, in order to examine the effect of multiple freeze‐thaw cycles and temperature fluctuation (i.e., taken out of the fridge before experimentation), but also to account for the instabilities they might cause, we utilized two different experimental regimes, comparing single‐batch and multi‐batch NPs for up to 4 weeks (Figure S1B, Supporting Information). This is, to our knowledge, the first work to date to extensively investigate medium‐term stability and directly compare the two NP technologies.

## 2 Results

As mentioned above, 2 different experimental regimes were employed in order to test both types of nanocarriers. This not only helped to verify findings, but also highlighted the effects of handling and multiple freeze‐thaw cycles on the NPs. To that end, the effect of temperature and cryoprotectants on NP stability was assessed every 2 weeks, for up to a month, and the best storage conditions were determined.

### 2.1 The Effect of Storage Conditions on Physicochemical Properties

**Figure 1:**
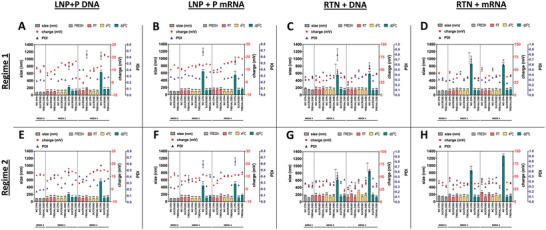
Physicochemical properties (size, charge, PDI) of LNPs and RTNs, freshly prepared and after 2 or 4 weeks of storage in different cryoprotectants and temperatures. A–D) Regime 1 nanoparticles, E–H) Regime 2 nanoparticles; (A, E) LNPs + DNA, (B, F) LNPs + mRNA, (C, G) RTNs + DNA, (D, H) RTNs + mRNA. Measurements were performed in triplicates for every condition (N = 3). Bar graphs represent the size, red squares (□) indicate the charge, and blue triangles (∆) represent the PDI. Results represent mean ± SEM. Statistical significance is displayed only for size. ns = not significant; *p < 0.05; **p < 0.01; ***p < 0.001.

First, the physicochemical properties of fresh nanoparticles and nanoparticles stored for 2 and 4 weeks were evaluated. These include their size, charge, and polydispersity index (PDI; an index of the homogeneity of an NP solution; PDI < 0.4 indicates a largely homogenous solution^[^ ^54^, ^55^ ^]^ (**Figure** **1**). Overall, even though for most conditions there were statistically significant differences between the freshly made and stored NPs, which in regime 2 could be attributed to batch‐to‐batch variability, all three NP characteristics rarely exceeded “physiological” values (Figure 1, Figures S2 and S3, Supporting Information) (size ≤ 200 nm; PDI < 0.4; charge = near‐neutral for LNPs, and strongly cationic for RTNs). Nevertheless, for both types of NPs and nucleic acid (NA) cargo, the nanoparticles stored at −80 °C without the protection of CPAs, demonstrated a significant (*p* < 0.001) increase in size and PDI after week 2, in both regimes. Remarkably, however, even though the size of the NPs changed, their respective charge remained almost the same throughout the storage conditions (Figure 1), with the exception of NPs stored at RT for 4 weeks, most notably LNPs, which demonstrated decreased charge values, possibly signifying being structurally compromised and the breakdown of nanoparticles and the outflow of negatively charged NAs in the solution.

**Figure 2:**
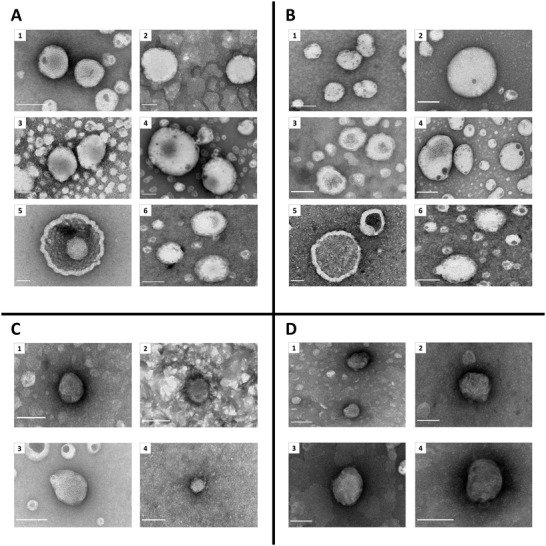
Negative staining transmission electron microscopy (TEM) imaging of LNPs and RTNs freshly prepared, and after 4 weeks in a variety of storage conditions. A) LNPs + DNA, B) LNPs + mRNA; (1) Fresh, (2) Week 4, sucrose at room temperature, (3) Week 4, no CPA at 4 °C, (4) Week 4, sucrose at 4 °C, (5) Week 4, no CPA at −80 °C, (6) Week 4, trehalose at −80 °C. C) RTNs + DNA, D) RTNs + mRNA; (1) Fresh, (2) Week 4, sucrose at room temperature, (3) Week 4, sucrose at −80 °C, (4) Week 4, trehalose at −80 °C. All scale bars represent a distance of 100 nm.

Next, negative staining transmission electron microscopy (TEM) was used in order to verify the dynamic light scattering (DLS) size measurements and visualize the morphology of NP formulations (**Figure** **2**). Here, some characteristic examples of regime 2 NPs are presented, freshly prepared and after 4 weeks. As seen in Figure 2, the TEM images are consistent with previous measurements regarding the size of the NPs. Furthermore, it is clear that they maintain their complex multilayer morphology and circular shape in most storage conditions, even though the number of intact nanoparticles varied greatly, with 4 °C sucrose (Figure 2A_4_,B_4_) and −80 °C trehalose (Figure 2A_6_,B_6_) presenting larger numbers. Moreover, several nanocomplexes, especially LNPs containing mRNA, had noticeable dark spots on their surface (Figure 2B). These could be caused by the accumulation of negative stain inside the indentations of the outer NP layers; however, another possible explanation could be the slow disintegration of the nanocomplexes, which leaves their interior components exposed. In contrast, both DNA and mRNA‐loaded LNPs stored at −80 °C in the absence of CPAs, appear to have grown in size immensely, demonstrating the characteristic “donut” morphology (Figure 2A_5_,B_5_) of empty or heavily disintegrated nanoparticles. RTNs were generally harder to image, likely due to their highly cationic nature, which attracts a larger quantity of the negative stain. Nevertheless, the RTNs we managed to image, although low in numbers, appear to preserve their morphology and size, even after 1 month (Figure 2C,D).

### 2.2 The Effect of Storage Conditions on Encapsulation Efficiency

**Figure 3:**
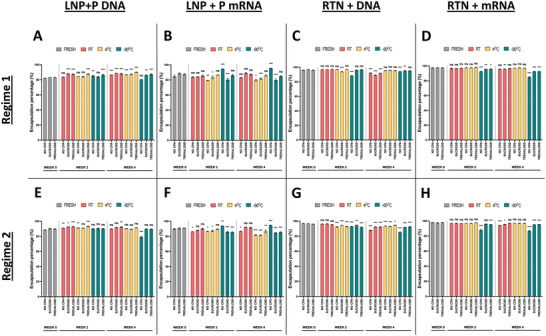
%Encapsulation efficiency of LNPs and RTNs, freshly prepared and after 2 or 4 weeks of storage in different cryoprotectants and temperatures. A–D) Regime 1 nanoparticles, E–H) Regime 2 nanoparticles; (A, E) LNPs + DNA, (B, F) LNPs + mRNA, (C, G) RTNs + DNA, (D, H) RTNs + mRNA. Measurements were performed in triplicates for every condition (N = 3). Bar graphs represent %encapsulation. Results represent mean ± SEM. ns = not significant; *p < 0.05; **p < 0.01; ***p < 0.001.

After preparing the LNP and RTN formulations and determining their physicochemical characteristics, their ability to encapsulate and retain nucleic acids in their interior for up to 4 weeks, was evaluated (**Figure** **3**). Overall, results were consistent across both regimes, and followed similar patterns with our physicochemical observations. In terms of entrapment, LNPs proved to be superior with encapsulation efficiencies reaching 98%, while RTNs managed to achieve an average efficiency of 87%. Furthermore, while studying encapsulation efficiency over time, even though statistically significant differences were observed, the majority of the formulations managed to maintain high levels of entrapment after one month, with the exception of unprotected NPs stored at −80 °C, which presented the largest decrease in encapsulation when compared to their initial (fresh) values, for all the LNP and DNA‐loaded RTN formulations (e.g., in regime 2: LNP + pDNA, 96.8% → 85.0%, *p* < 0.001; LNP + mRNA, 98.0% → 86.6%, *p* < 0.001; RTN + pDNA, 89.7% → 79.0%, *p* < 0.001). Interestingly, regime 1 LNPs + DNA (Figure 3A) and RTNs carrying the luciferase mRNA (Figure 3D,H) presented an increase in encapsulation for this same storage condition, in contrast to our other measurements, an effect likely caused by the degradation of unencapsulated DNA and mRNA.

**Figure 4:**
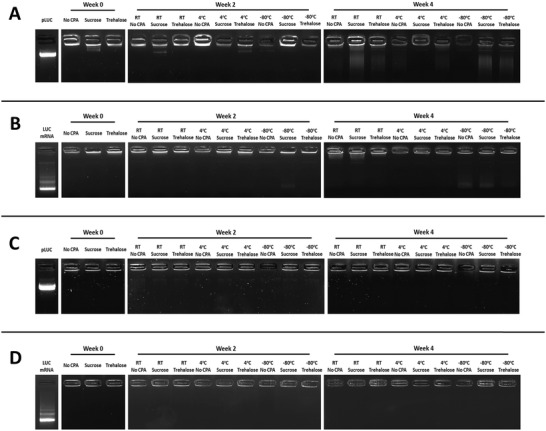
Gel retardation assay of LNPs and RTNs, freshly prepared and after 2 or 4 weeks of storage in different cryoprotectants and temperatures. A) LNPs + DNA, B) LNPs + mRNA, C) RTNs + DNA, D) RTNs + mRNA. 250 ng of DNA or mRNA‐carrying nanoparticles were loaded in each well. Bands in the wells represent intact NPs.

Following the encapsulation assay results, and in order to verify our findings, a gel retardation assay was carried out. This assay relies on the fact that encapsulated nucleic acids will not be able to migrate freely through agarose during gel electrophoresis, in contrast to the unencapsulated NAs. Consequently, encapsulated nucleic acids and thus the entrapment ability of our NPs can be easily visualized with this method (**Figure** **4**). In total, almost all the tested formulations demonstrated a good encapsulation efficiency during the gel retardation assay, confirming our previous findings. Moreover, once again we consistently observe a decrease (Figure 4B,D), or outright absence (Figure 4A,C), in the amount of encapsulated NAs in both LNPs and RTNs stored at −80 °C without cryoprotectants, signaling the instability and loss of function of these formulations.

### 2.3 The Effect of Storage Conditions on Transfection Efficiency

**Figure 5:**
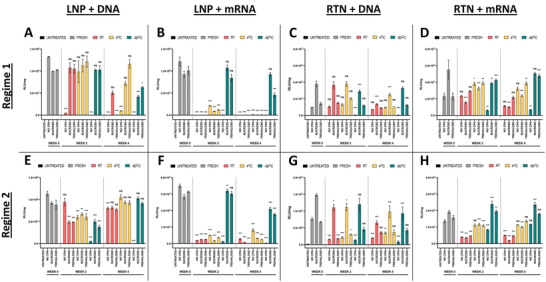
Luciferase expression in HEK‐293 cells 24 h after transfection with LNPs and RTNs, freshly prepared and after 2 or 4 weeks of storage in different cryoprotectants and temperatures. A–D) Regime 1 nanoparticles, E–H) Regime 2 nanoparticles; (A, E) LNPs + DNA, (B, F) LNPs + mRNA, (C, G) RTNs + DNA, (D, H) RTNs + mRNA. Measurements were performed in sextuplicates for every condition (N = 6). Bar graphs represent relative light units per mg of total protein (RLU mg^−1^). Results represent mean ± SEM. ns = not significant; *p < 0.05; **p < 0.01; ***p < 0.001.

Having demonstrated that both LNPs and RTNs retain their physicochemical characteristics and NA entrapment efficiencies for most of the storage conditions, for up to 4 weeks, we decided to investigate if this would also apply to their ability to efficiently transfect cells in vitro (**Figure** **5**). Even though it has been previously established that both NP technologies can deliver RNA molecules in vitro, with high efficacy [siRNA,^[^ ^42^, ^44^, ^46^, ^56^, ^57^ ^]^ mRNA^[^ ^7^, ^58^ ^]^], this is the first time we are extensively testing these formulations for their stability, and the first time we are investigating the delivery of DNA by LNPs. To that end, and in order to further validate our results, we utilized HEK‐293 cells, an epithelial cell line that was isolated from the kidney of a human embryo. This cell line was chosen as it is known to demonstrate fast growth and ease of transfection.

As shown in Figure 5, the storage conditions, the type of the nanoparticles, as well as the nature of the nucleic acid cargo, greatly affect the medium‐term stability of the nanocomplexes, and hence their transfection efficiency. Overall, our findings stay consistent between the two regimes. However, nanoparticles in regime 1 appear to lose their efficiency faster across all conditions, when compared to nanoparticles in regime 2, suggesting that the handling of the nanocomplexes (multiple analyses every 2 weeks) and the multiple freeze‐thaw cycles of regime 1, have some effect on their stability, especially when stored under less than ideal conditions.

In detail, nanoparticles stored at 4 or −80 °C in the presence of cryoprotectants, with the exception of mRNA‐loaded LNPs, were able to maintain their transfection efficiency, leading to potent luciferase expression, even after one month. Looking at the cryoprotectants, sucrose achieved greater levels of protection than trehalose, in accordance with other studies,^[^ ^26^, ^37^, ^59^ ^]^ and even appears to have increased the efficiency of RTNs (Figure 5C,D,G), likely by further stabilizing the nanocomplexes. It is also worth noting, that the LNPs carrying the luciferase mRNA proved to be extremely sensitive to the storage conditions, losing almost all of their potency when stored outside −80 °C, after just 2 weeks. The rest of the formulations retained a good level of their efficiency in all three temperatures during the first two weeks of storage, even without the presence of a cryoprotectant. However, by the end of week 4, mRNA‐loaded NPs stored at RT and unprotected NPs stored at −80 °C, lost it almost completely, suggesting that their structural integrity, and hence the protection offered to their NA cargos was compromised.

**Figure 6:**
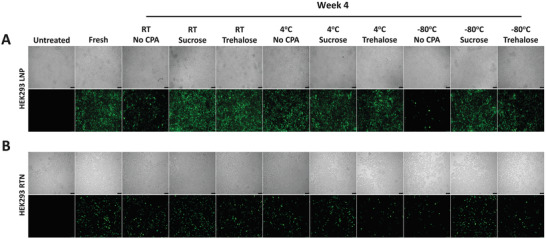
Brightfield (grayscale) and fluorescent (green) imaging of GFP expression in HEK‐293 cells 24 h after transfection with LNPs and RTNs, freshly prepared and after 4 weeks of storage in different cryoprotectants and temperatures. A) LNPs + EGFP pDNA, B) RTNs + EGFP pDNA.

Subsequently, LNPs and RTNs containing a plasmid coding for the GFP protein were formulated, and were used to transfect HEK‐293 cells immediately after formulation and after 4 weeks of storage in a variety of conditions, in order to visualize and further validate their transfection efficiencies, or lack thereof (**Figure** **6**).

Figure 6 shows that there is a clear difference between the transfection efficiency of LNPs (Figure 6A) versus that of the RTNs (Figure 6B), with the former significantly outperforming the latter. Moreover, we can notice an almost perfect correlation between GFP and luciferase transfection results, at least for LNPs, which further reinforces our previous observations. To elaborate, when stored at −80 °C, unprotected LNPs lose their ability to transfect their target cells almost completely, in accordance with the physicochemical and encapsulation data. Notably, these results also demonstrate that unprotected LNPs stored at RT, as well as at −80 °C in the presence of 12% w/v trehalose, are starting to experience a decrease in their efficiencies, 1 month after formulation, but still retain some of their functionality.

Similarly, even though it is harder to distinguish differences in GFP expression, as a result of the lower efficiency of the RTNs compared to the LNPs, these results nonetheless follow the same patterns, with −80 °C no CPA failing to transfect most of the cells. Interestingly, RTNs stored in the cryoprotectant trehalose display lower functionality in all 3 temperatures, in contrast to RTNs stored in sucrose, once again validating the luciferase data.

### 2.4 Cell Viability

**Figure 7:**
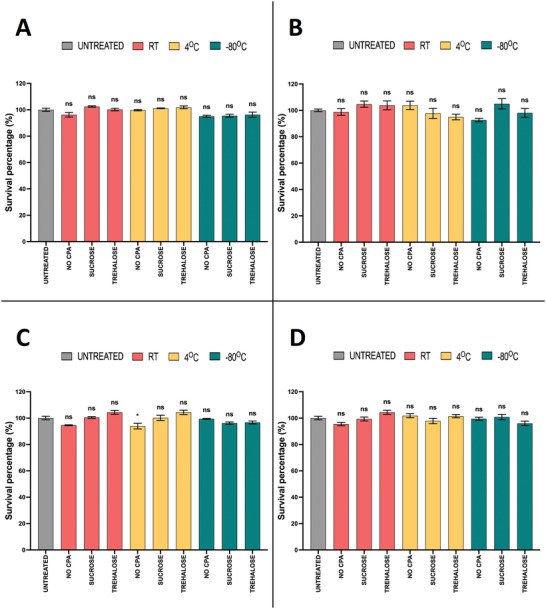
Cell viability of HEK‐293 cells, 24 h after transfection with LNPs and RTNs stored in different cryoprotectants and temperatures for 2 weeks. A) LNPs + DNA, B) LNPs + mRNA, C) RTNs + DNA, D) RTNs + mRNA. The fluorescent variant of the resazurin cell viability assay was used. Measurements were performed in triplicates for every condition (N = 3). Bar graphs represent %survival. Results represent mean ± SEM. ns = not significant; *p < 0.05.

Last, in order to assess if our formulations were cytotoxic in vitro and determine whether the different cryoprotectants and storage conditions affect the safety profiles of the nanoparticles, we performed the resazurin cell viability assay with NPs stored in a variety of conditions, for 2 weeks (**Figure** **7**).

In summary, all formulations tested (LNPs + DNA, LNPs + mRNA, RTNs + DNA, RTNs + mRNA) proved to be non‐cytotoxic (p > 0.05) in vitro, in accordance with previous experiments using fresh NPs,^[^ ^44^, ^46^ ^]^ except RTNs + DNA stored at 4 °C without a CPA (Figure 7C), which slightly decreased cell viability to 93.9% (p = 0.04), an otherwise acceptable level. These data not only demonstrate that any decrease in transfection efficiency we observed was not a result of cytotoxicity and cell death, but it also substantiates that both LNP and RTN technologies produce safe, well‐accepted nanoparticles, while also offering very high transfection efficiencies, as demonstrated with LNPs.

## 3 Discussion

Nucleic acid delivery systems, such as lipid‐based nanoparticles, are showing great promise for the treatment of a variety of diseases, including a range of tumors^[^ ^60^, ^61^ ^]^ and COVID‐19,^[^ ^6^, ^21^ ^]^ and are considered the future of nanomedicine and novel therapeutics.^[^ ^62^ ^]^ However, their stability over time and the effects that different storage conditions have on it, are not thoroughly studied and understood yet, as it became clear during the beginning of the COVID‐19 pandemic.^[^ ^63^ ^]^ It has been demonstrated before, that nanoparticles undergo mechanical stress and chemical modifications during storage,^[^ ^24^, ^25^ ^]^ especially during freezing conditions, that can lead to their aggregation and fusion, and can potentially influence the fate of the NPs by affecting their physicochemical properties and ability to deliver their cargo.^[^ ^59^, ^64^, ^65^ ^]^ Hence, understanding and optimizing the stability of lipid‐based delivery systems has become a priority, as it will eventually improve their clinical potential by increasing their shelf‐life and safety, and minimizing the costs and variability of their production.

In this study, we demonstrate that both LNPs and RTNs are able to maintain most of their transfection efficiency under a range of storage conditions, spanning various temperatures and cryoprotectants, for up to 4 weeks in aqueous media. Loss of function occurred at different conditions and time points for every formulation, with distinct patterns emerging between DNA and mRNA NPs, suggesting that NP stability is closely associated with their cargo, with DNA‐loaded nanocomplexes remaining stable in a wider variety of storage conditions than mRNA‐loaded ones.

However, irrespective of nucleic acid cargo, experimental regime, or type of nanoparticle, NPs stored at −80 °C without cryoprotectants (−80 °C, no CPA) suffered the biggest decline in efficiency, starting in week 2. This decrease corresponds with the physicochemical measurements, where at −80 °C, no CPA‐stored NPs demonstrated significant (*p* < 0.001) increase in size and PDI invariably, indicating the aggregation of the nanoparticles and the fusion of their membranes under the freezing conditions, which in turn can result in the formation of clusters that span several microns in size.^[^ ^66^ ^]^ Relatedly, when studied under TEM, the LNPs stored at −80 °C without CPA were bigger in size and demonstrated a characteristic “donut” morphology (Figure 2A_5_,B_5_), caused by the collapse of the nanoparticles due to loss of their structural integrity, in accordance with our other observations.

Interestingly, even though the correlation between the physicochemical properties of nanoparticles and their transfection efficiency and cytotoxicity is well established for many types of NPs,^[^ ^48^, ^67^, ^68^, ^69^ ^]^ this was not the case in our study, as well as other studies of groups working with similar lipid‐based nanocomplexes.^[^ ^22^, ^70^ ^]^ To elaborate, for all storage conditions other than at −80 °C with no CPA, very small or no changes in NP size, charge, and PDI were observed that could indicate the breakdown or destabilization of our formulations (Figure 1). Nevertheless, when we evaluated the transfection efficiencies of these same complexes, we were surprised to discover that many of the formulations had lost their ability to efficiently transfect cells in vitro (Figure 5). This could be attributed to chemical transformations on the surface of the nanoparticles, like hydrolysis of the ester bonds of the lipids,^[^ ^22^, ^71^ ^]^ or destabilization and degradation of their nucleic acid cargo due to higher temperatures. In the case of LNPs containing mRNA, it has been suggested that destabilization of the nanocomplexes can lead to the production of electrophilic impurities, derived from the ionizable cationic lipid component of the LNPs, which can disrupt the mRNA cargo and negatively impact its translational ability,^[^ ^72^ ^]^ explaining the loss of function in almost all conditions, starting at week 2. In addition, the electrophilic impurities could also explain our TEM observations of dark spots on the surface of LNP + mRNA particles (Figure 2B), indicating the compromise of the integrity of the lipid membrane.

Following the loss of potency of some formulations, the encapsulation ability of the nanoparticles was investigated further by utilizing the RiboGreen and PicoGreen assays. Interestingly, even though most formulations appeared to maintain high encapsulation efficiencies, a common entrapment pattern emerged, that was more prevalent in the RTN formulations, likely due to their lower encapsulation efficiency and higher instability. Interestingly, for what we could consider the most unstable storage conditions, entrapment levels increased over time, even surpassing the initial measurements. This conflicting increase has been observed before^[^ ^22^ ^]^ and it may have been due to the degradation of the unencapsulated nucleic acids during the storage period and the manner in which encapsulation efficiency is calculated. The degradation of the unencapsulated nucleic acids could also explain the absence of extra bands and the lower intensity of the existing bands observed in most wells of the gel retardation assay. According to the literature,^[^ ^22^ ^]^ if stored for longer periods of time, we would inevitably observe the expected gradual decrease in encapsulation efficiency after one month.

Finally, in addition to determining the best storage conditions and the stability of our formulations, we directly compared two similar, but very different, types of nanoparticles, in terms of potency, entrapment efficiency, safety, and stability. In all the aforementioned categories, LNPs proved to be exceptionally better than liposomes (RTNs), demonstrating excellent encapsulation and transfection efficiencies, while also being non‐cytotoxic and stable for at least one month, thus, establishing themselves as superior nanocarriers for the delivery of therapeutics.

The addition of the cryoprotectants sucrose and trehalose, mitigated the damage sustained by the NPs due to the storage conditions, and even improved the transfection efficiency of the RTNs. Although the exact mechanism of how sugars, and especially disaccharides, are able to stabilize and preserve nanoparticles in freezing conditions is contested,^[^ ^26^, ^37^ ^]^ there have been many studies over the years highlighting their cryoprotective effects and their value in industry.^[^ ^48^, ^64^, ^73^, ^74^, ^75^, ^76^ ^]^ Most recently, the companies producing the LNP‐based, mRNA vaccines for COVID‐19 utilized sucrose for the preservation of their formulations, aligning with our observations, and those of others, that suggest its cryoprotective effects are superior to those of trehalose and other similar CPAs.^[^ ^26^, ^37^, ^59^ ^]^

Throughout this study, we demonstrated that storage of our lipid‐based nanoparticles at −80 °C in a 12% w/v sucrose solution, provided the best results in terms of preservation of their properties and characteristics for at least 4 weeks, regardless of nucleic acid cargo or type of nanoparticle. The next step of this research is to develop and optimize the lyophilization of our nanocomplexes, in order to evaluate their stability for longer periods of time and compare between the different physical states (i.e., in solution or lyophilized powder). Future work will also focus on testing more of our novel formulations, in an effort to better understand how the different components, molecular weight and N/P ratios, and cryoprotectants can improve or hinder the stability of the nanoparticles.

Importantly, we demonstrated here for the first time that LNPs containing the ionizable lipid CL4H6 are able to efficiently encapsulate and deliver plasmid DNA in vitro, leading to potent protein expression, as shown by the GFP experiments (Figure 6A). LNPs have been proven to be very successful and have found many uses for the delivery of mRNAs and siRNAs,^[^ ^1^, ^10^, ^11^, ^21^, ^77^ ^]^ however, viral vectors are still monopolizing the field of gene therapy and the delivery of DNA therapeutics^[^ ^78^ ^]^ regardless of their disadvantages, due to lack of better alternatives. Therefore, the results presented in this study not only exemplify the versatility and potential of CL4H6‐LNPs as non‐viral vectors, but could also signify the shift of LNPs, from carriers of RNA therapeutics, where they have proven their value, to the gene therapy field, where non‐viral formulations are still not efficient.

## 4 Experimental Section

### Materials

The pH‐sensitive cationic lipid CL4H6 was made in house as previously described.^[^ ^79^ ^]^ The lipids 1,2‐dioleoyl‐sn‐glycero‐3‐phosphoethanolamine (DOPE), 1,2‐di‐O‐octadecenyl‐3‐trimethylammonium propane (chloride salt) (DOTMA), and 1,2‐dimyristoyl‐rac‐glycero‐3‐methoxypolyethylene glycol‐2000 (DMG‐PEG_2000_), as well as the disaccharides sucrose and trehalose were purchased from SIGMA Aldrich (St. Louis, MO). LDL receptor (LDLR) targeting peptide AT‐19 (K16RVRRGACMPRLRGCLG)^[^ ^80^ ^]^ was synthesized by China Peptides (Suzhou, China). The Quant‐it RiboGreen and Quant‐it PicoGreen Assay kits were purchased from Thermo Fisher Scientific (Loughborough, UK). The gWiz plasmid containing the luciferase gene was purchased from Aldevron (Madison, WI). The CleanCap FLuc mRNA (5moU) was purchased from Trilink Biotechnologies (San Diego, CA). The EGFP‐N1 plasmid was obtained from Addgene (Watertown, MA). The lipid structures, peptide sequence, and nucleic acids used are shown in **Table** **1**.

**Table 1:** Structures of the different lipids and cryoprotectants and sequences of the peptides and nucleic acids

Function	Lipids	Structure
Lipid	7‐(4‐(dipropylamino)butyl)‐7‐hydroxytridecane‐1,13‐diyl dioleate (CL4H6)	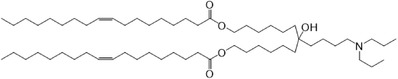
Lipid	1,2‐dioleoyl‐sn‐glycero‐3‐phosphoethanolamine (DOPE)	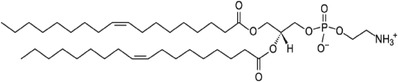
Lipid	1,2‐di‐O‐octadecenyl‐3‐trimethylammonium propane (chloride salt) (DOTMA)	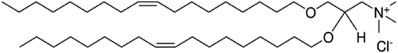
Lipid	1,2‐dimyristoyl‐rac‐glycero‐3‐methoxypolyethylene glycol‐2000 (DMG‐PEG _2000_)	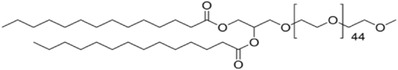
**Function**	**Peptides**	**Sequence**
LDLR Targeting	Peptide AT‐19	K_16_–RVRR–GA–CMPRLRGC–LG
**Function**	**Nucleic Acids**	**Product**
Protein Expression	gWiz Luciferase Mammalian Expression Vector – pDNA	Luciferase
Protein Expression	CleanCap FLuc mRNA (5moU) – modified mRNA	Luciferase
Protein Expression	EGFP‐N1 Vector – pDNA	GFP
**Function**	**Cryoprotectants**	**Structure**
Cryoprotection	Sucrose	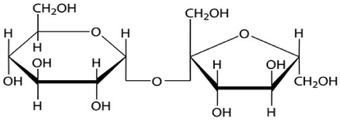
Cryoprotection	Trehalose	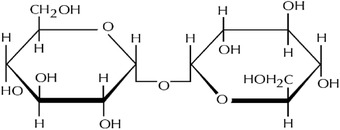

### Nanoparticle Formulations – LNPs

CL4H6, DOPE, and DMG‐PEG_2000_ were prepared in 90% t‐BuOH at a final volume of 400 µL and a molar ratio of 50:50:1. 100 µL of DNA or mRNA (0.4 mg mL^−1^) were mixed with the lipids to give an N/P ratio of 6.25. Next, the LNPs were prepared in 20 mm MES buffer (pH 6.0) using the alcohol dilution method, and subsequently, the residual alcohol was removed and the sample was concentrated via ultrafiltration, as previously described.^[^ ^46^ ^]^ Then the resulting LNPs were mixed with peptide AT‐19 at a weight ratio of 4:1 (peptide: nucleic acid) by rapid mixing to obtain the final nanoparticles.

### Nanoparticle Formulations – RTNs

DOTMA and DOPE were mixed at a molar ratio of 1:1 and liposomes were prepared by the thin‐film hydration method.^[^ ^81^ ^]^ RTN formulations were prepared by mixing the DOTMA/DOPE liposomes with peptide AT‐19 and DNA in pre‐established ratios (1:4:1 – L:P:D) or mRNA (3:4:1 – L:P:R) to produce RTNs.

### Nanoparticle Storage at Various Temperatures with Different Cryoprotectants

The LNPs and RTNs were formulated as described above, and then diluted either in 1X PBS, a 12% w/v sucrose solution—sterile filtered, or a 12% w/v trehalose solution—sterile filtered. Next, the nanoparticles containing PBS, or each of the cryoprotectants, were equally split, and aliquots were stored in different conditions (room temperature, 4 or −80 °C), for up to a month (Figure S1A, Supporting Information).

### Experimental Regimes

In regime 1 (R1), a large batch of NPs was prepared at the beginning of the experiment (Day 0), was equally divided between the different CPAs, and their physicochemical characteristics and efficacy were measured, before storing them in the various aforementioned conditions. Every 2 weeks (Day 14 and Day 28), these NPs were taken out of storage and had their stability evaluated (Figure S1B, Supporting Information). Conversely, in regime 2 (R2), a new, smaller batch of NPs was prepared every 2 weeks, for up to one month, and the formulations were subsequently divided between different CPAs and stored under different conditions. After 4 weeks, all the previously prepared NPs (14 and 28 days of storage), as well as freshly prepared NPs (Day 0), had all their properties measured concurrently, and their stability evaluated (Figure S1B, Supporting Information).

### Nanoparticle Size and Zeta Potential

A Nano ZS Zetasizer (Malvern Instruments, Malvern, UK) was used to measure the size and zeta potential of the nanoparticles by dynamic light scattering (DLS) and laser Doppler anemometry, respectively, as previously described.^[^ ^82^ ^]^ Measurements were performed in triplicate for each sample and the results were analyzed using the built‐in software (DTS version 7.12).

### Transmission Electron Microscopy (TEM)

NPs were applied on glow‐discharged carbon‐coated copper grids (300 mesh), stained with 1% w/v uranyl acetate in water, and blotted with Whatman #1 filter paper. After complete drying, samples were observed with FEI (US) Tecnai 12 BioTwin Transmission Electron Microscope at 80 kV acceleration voltage. Images were acquired with a Gatan Orius SC1000A CCD bottom‐mounted camera.

### Encapsulation Assay

The mRNA encapsulation efficiency was determined using the Quant‐it RiboGreen assay. 100 µL of nanoparticles or mRNA solution (for standard curve) were diluted in 100 µL of 10 mm HEPES buffer (pH 7.4) containing RiboGreen, in the presence or absence of 20 µg mL^−1^ dextran sulfate and 0.1% w/v Triton X‐100, and were then added to a 96‐well plate (Falcon, Fisher Scientific UK). Fluorescence was measured using a FLUOstar Omega plate reader (BMG Labtech, Aylesbury, UK). Measurements were performed in triplicate for each sample and the mRNA concentrations were determined in reference to an mRNA standard curve. The mRNA encapsulation efficiency was then calculated by the following formula:

(1) $$\begin{array}{ccc} & & \mathrm{mRNA}\;\mathrm{encapsulation}\;\%\\ & & \;=\frac{\mathrm{Encapsulated}\;\mathrm{mRNA}-\mathrm{Unencapsulated}\;\mathrm{mRNA}}{\mathrm{Total}\;\mathrm{mRNA}\;\mathrm{concentration}}\times 100 \end{array}$$

The same protocol was used to determine the DNA encapsulation efficiency by substituting Quant‐it RiboGreen with Quant‐it PicoGreen and using as a reference a DNA standard curve.

### Gel Retardation Assay

The gel retardation assay was used to assess the nucleic acid binding ability of the nanoparticles. 250 ng of LNPs and RTNs, containing luciferase DNA or mRNA, were loaded in each well of a 1% agarose gel, made in Tris‐acetate‐EDTA (TAE) buffer, stained with 1 µg mL^−1^ ethidium bromide, and electrophoresed at a voltage of 80 V for 30 min with TAE as the running buffer, in order to test whether the nucleic acids remained encapsulated. Naked luciferase pDNA or mRNA were used as controls, respectively. The results were then visualized under a UV illuminator (Bio‐Rad Laboratories, CA, USA).

### Cell Culture

In vitro transfections were performed using the HEK‐293 cell line, which was originally obtained from the American Type Culture Collection (ATCC). Cells were cultured in humidified 5% CO_2_, at 37 °C, in Dulbecco's Modified Eagle Medium (DMEM) (Gibco) supplemented with 10% fetal calf serum and 2 mmol L^−1^ L‐glutamine. Passages 10–30 were used for the experiments.

### In Vitro Transfection

HEK‐293 cells were seeded in clear bottom 96‐well plates (Corning, UK) at a density of 5 × 10^4^ cells per well and were incubated for 24 h to reach approximately 60% – 70% confluency before the LNPs or RTNs were added. For the transfections, 250 ng well^−1^ of pDNA or 100 ng well^−1^ of mRNA were used. RTNs were diluted in optiMEM (Gibco) to a total volume of 100 µL well^−1^ before they were added to the cells. After 4 h, 50 µL of complete media were added to each well. LNPs were diluted in complete media to a total volume of 150 µL well^−1^ since the beginning, thus requiring no media addition after 4 h. The cells were then incubated for 24 h at 37 °C before further analyses were performed.

### Microscopy

Cells were washed with 1X PBS 24 h following transfection with LNPs or RTNs containing the EGFP‐N1 plasmid, and observed using a Leica DMi8 fluorescent microscope (Leica, Germany), without further processing of the samples. Brightfield images were acquired using a Leica DMC4500 camera, whereas fluorescent images were acquired by a Leica DFC365 FX CCD fluorescence camera. The 10X objective lens was used for photo acquisition.

### Cell Proliferation Assay

The Resazurin Assay Kit (Abcam, Cambridge, UK) was used to determine cell viability. Cells were washed with 1X PBS 24 h following transfection with LNPs or RTNs containing pDNA or mRNA, and 100 µL of fresh media were added in each well, as well as 100 µL of a 2X resazurin solution. The cells were then incubated at 37 °C for 4 h. Following this, fluorescence readings were recorded using a FLUOstar Omega plate reader (BMG Labtech, Aylesbury, UK) set at 550 nm excitation and 590 nm emission. The cell viability was calculated as a percentage of the viability of the control untreated cells. Triplicate wells were used and measured for each condition.

### Luciferase Assay

Cells were washed with 1X PBS 24 h following transfection with LNPs or RTNs containing Luciferase plasmid or mRNA, and lysed with 20 µL Reporter Lysis Buffer (Promega, Southampton, UK) by incubation at 4 °C for 20 min, then −20 °C for another 20 min, and finally −80 °C for at least 45 min, followed by thawing at room temperature. Luciferase activity was assessed using the Luciferase Assay System (Promega, Southampton, UK) on a FLUOstar Omega plate reader (BMG Labtech, Aylesbury, UK). The results were standardized for protein content using the Bradford protein assay (Bio‐Rad, Hertfordshire, UK) and expressed as relative luminescence units per mg of protein (RLU mg^−1^). Sextuplicate wells were used and measured for each condition.

### Statistical Analysis

All data were subjected to one‐way ANOVAs with multiple comparisons followed by Bonferroni's post‐hoc correction. Samples were compared against freshly prepared NPs dissolved in each of the 3 CPAs. Values that were statistically significant were expressed as follows: *p < 0.05; **p < 0.01; ***p < 0.001. All graphs used in the paper display mean and standard error of the mean (SEM). Graph‐Pad Prism 8.4.2. was used for all the analyses and the generation of the graphs.

## Conflict of Interest

The authors declare no conflict of interest.

## Author Contributions

Conceptualization, A.D.T.; Methodology, K.N.K., N.P., E.M., Y.S., A.M., A.P., H.H., and A.D.T.; Formal analysis, K.N.K., K.X.T., and A.D.T.; Writing–original draft preparation, K.N.K.; Writing–review and editing, K.N.K., N.P., E.M., K.X.T., Y.S., A.M., A.P., P.J.W., H.H., C.Y., and A.D.T.; Supervision, C.Y. and A.D.T.; Project administration, A.D.T.; Funding acquisition, A.D.T. and C.Y.; All authors have read and agreed to the published version of the manuscript.

## Supporting information

Supporting Information

## Data Availability

The data that support the findings of this study are available from the corresponding author upon reasonable request.
