# Supplementary material for: The Effect of Cryoprotectants and Storage Conditions on the Transfection Efficiency, Stability, and Safety of Lipid‐Based Nanoparticles for mRNA and DNA Delivery
Source: Adv Healthc Mater. 2023 Mar 21;12(18):2203022. doi: 10.1002/adhm.202203022 (PMC11468535; doi:10.1002/adhm.202203022)
Supplement: Supplementary file 1 — Supporting Information [file ADHM-12-2203022-s001.pdf]

ADVANCED HEALTHCARE MATERIALS

Supporting Information

for *Adv. Healthcare Mater.*, DOI 10.1002/adhm.202203022

The Effect of Cryoprotectants and Storage Conditions on the Transfection Efficiency, Stability, and Safety of Lipid-Based Nanoparticles for mRNA and DNA Delivery

Konstantinos N. Kafetzis, Natalia Papalamprou, Elisha McNulty, Kai X. Thong, Yusuke Sato, Aleksandr Mironov, Atul Purohit, Philip J. Welsby, Hideyoshi Harashima, Cynthia Yu-Wai-Man and Aristides D. Tagalakis**

Supporting Information

The effect of cryoprotectants and storage conditions on the transfection efficiency, stability and safety of lipid-based nanoparticles for mRNA and DNA delivery

Konstantinos N. Kafetzis (ORCID: https://orcid.org/0000-0002-4958-345X), Natalia Papalamprou, Elisha McNulty, Kai X. Thong, Yusuke Sato, Aleksandr Mironov, Atul Purohit, Philip J. Welsby, Hideyoshi Harashima, Cynthia Yu-Wai-Man (ORCID: https://orcid.org/0000-0003-4868-5187) and Aristides D. Tagalakis (ORCID: https://orcid.org/0000-0002-4610-0803)**

Supplementary Figures

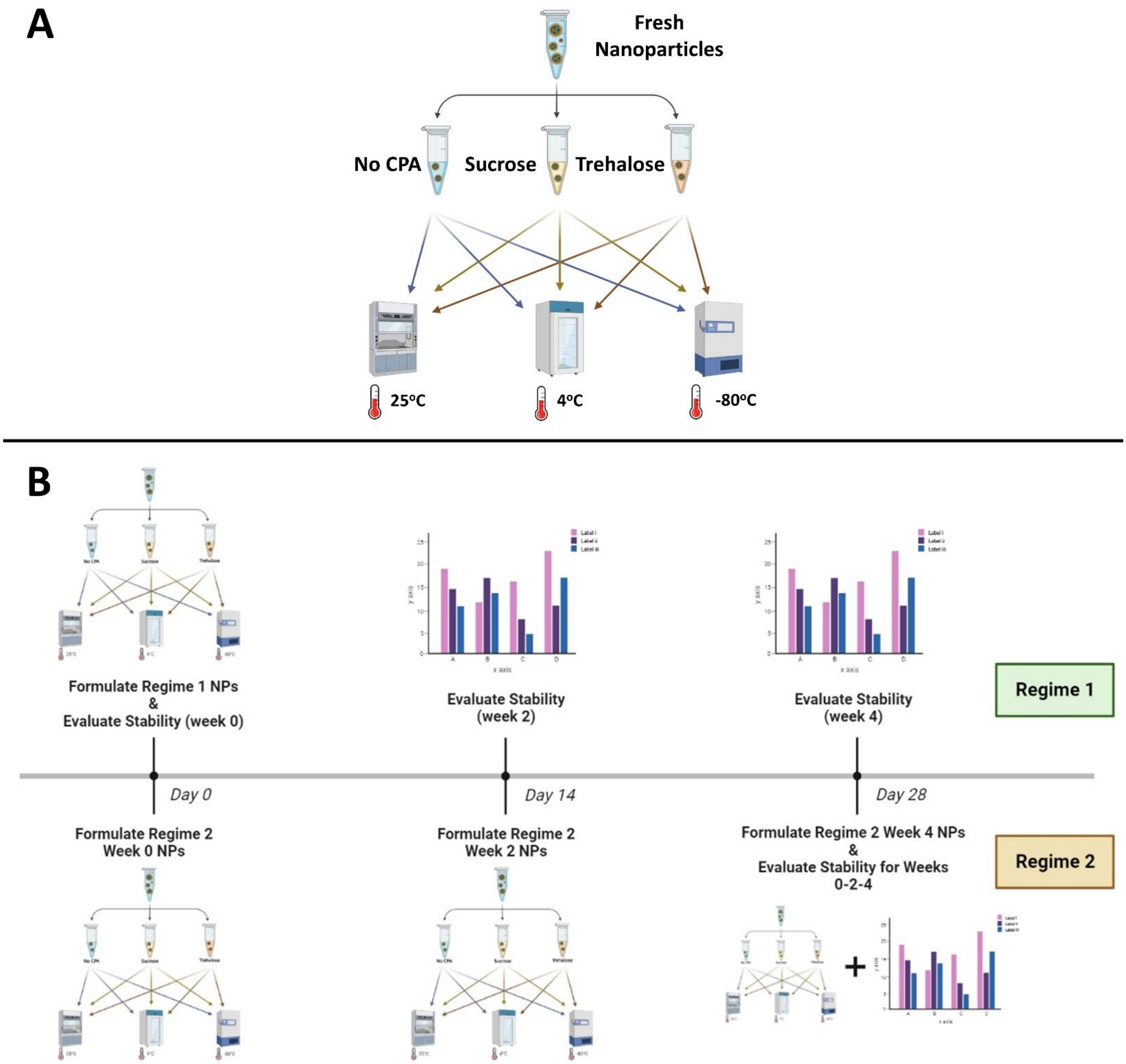

Figure S1. Schematic representation of the experimental regimes. (A) NPs were freshly prepared and then equally divided between the different CPAs and storage conditions, **(B)** Regime 1 NPs were prepared fresh in Day 0 and their stability was evaluated every 2 weeks (measurements at weeks 0, 2 and 4); In regime 2, fresh NP batches were prepared every 2 weeks and their stability was evaluated only on week 4.

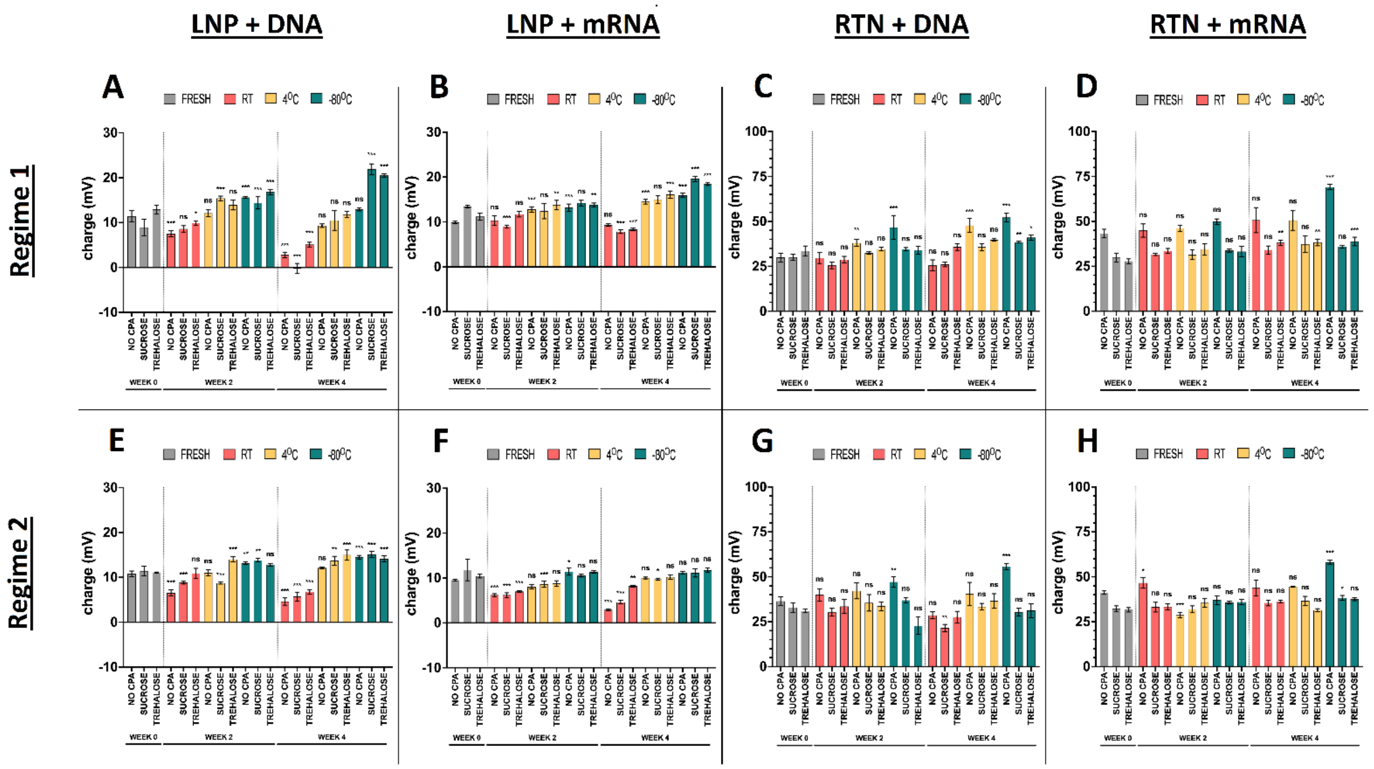

Figure S2. Surface charge (Zeta potential) of LNPs and RTNs, freshly prepared and after 2 or 4 weeks of storage in different cryoprotectants and temperatures. (A-D) Regime 1 nanoparticles, **(E-H)** Regime 2 nanoparticles; **(A, E)** LNPs + DNA, **(B, F)** LNPs + mRNA, **(C, G)** RTNs + DNA, **(D, H)** RTNs + mRNA. Measurements were performed in triplicates for every condition (N=3). Bar graphs represent the surface charge of the nanoparticles. Results represent mean ± SEM. ns = not significant; *, $p < 0.05$; **, $p < 0.01$; ***, $p < 0.001$.

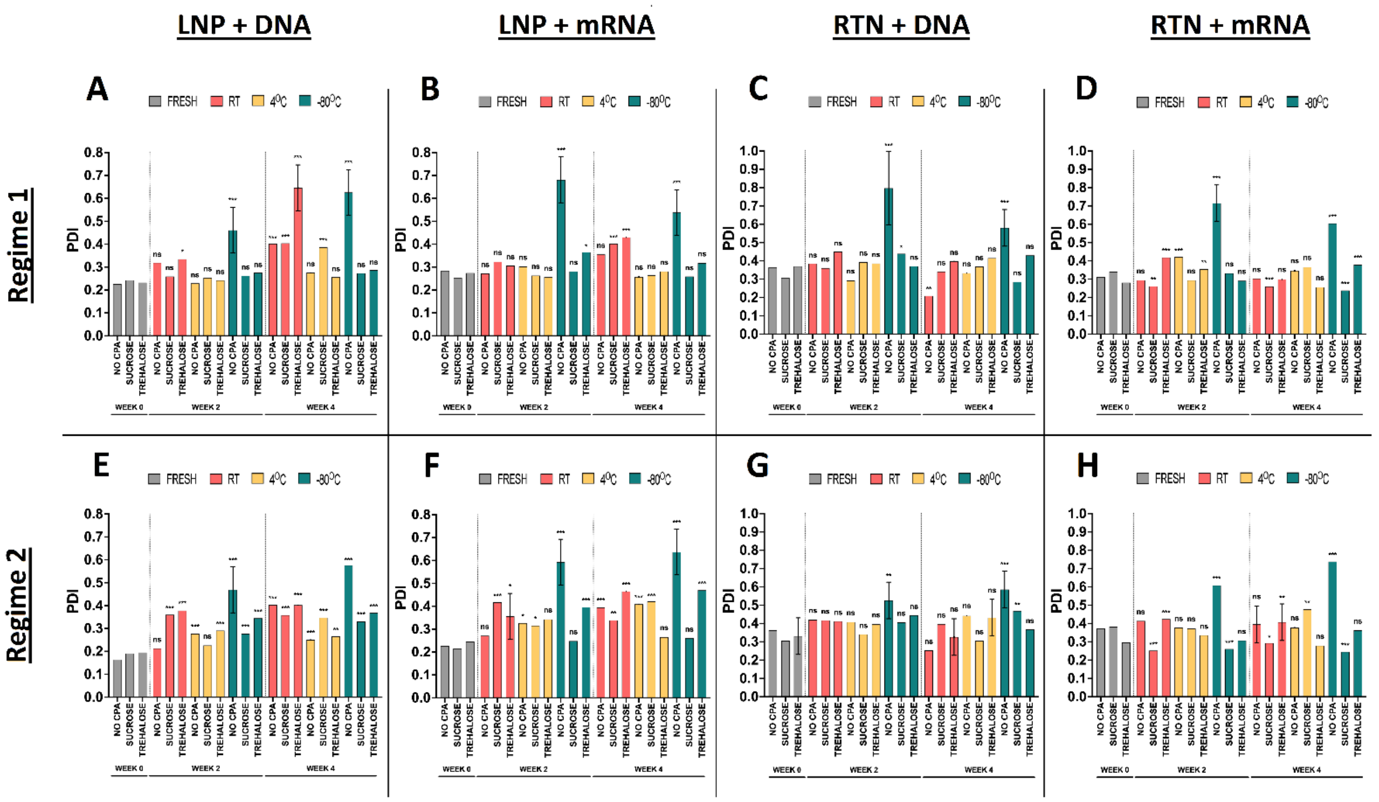

Figure S3. Polydispersity Index (PDI) of LNPs and RTNs, freshly prepared and after 2 or 4 weeks of storage in different cryoprotectants and temperatures. (A-D) Regime 1 nanoparticles, **(E-H)** Regime 2 nanoparticles; **(A, E)** LNPs + DNA, **(B, F)** LNPs + mRNA, **(C, G)** RTNs + DNA, **(D, H)** RTNs + mRNA. Measurements were performed in triplicates for every condition (N=3). Bar graphs represent the PDI of the nanoparticles. Results represent mean ± SEM. ns = not significant; *, $p < 0.05$; **, $p < 0.01$; ***, $p < 0.001$.
